# Physical Demand but Not Dexterity Is Associated with Motor Flexibility during Rapid Reaching in Healthy Young Adults

**DOI:** 10.1371/journal.pone.0127017

**Published:** 2015-05-13

**Authors:** Christian Greve, Tibor Hortobàgyi, Raoul M. Bongers

**Affiliations:** 1 Center for Human Movement Science, University of Groningen, University Medical Center Groningen, Groningen, The Netherland; 2 Department of Sport, Exercise and Rehabilitation, Northumbria University, Newcastle-upon-Tyne, United Kingdom; University of Surrey, UNITED KINGDOM

## Abstract

Healthy humans are able to place light and heavy objects in small and large target locations with remarkable accuracy. Here we examine how dexterity demand and physical demand affect flexibility in joint coordination and end-effector kinematics when healthy young adults perform an upper extremity reaching task. We manipulated dexterity demand by changing target size and physical demand by increasing external resistance to reaching. Uncontrolled manifold analysis was used to decompose variability in joint coordination patterns into variability stabilizing the end-effector and variability de-stabilizing the end-effector during reaching. Our results demonstrate a proportional increase in stabilizing and de-stabilizing variability without a change in the ratio of the two variability components as physical demands increase. We interpret this finding in the context of previous studies showing that sensorimotor noise increases with increasing physical demands. We propose that the larger de-stabilizing variability as a function of physical demand originated from larger sensorimotor noise in the neuromuscular system. The larger stabilizing variability with larger physical demands is a strategy employed by the neuromuscular system to counter the de-stabilizing variability so that performance stability is maintained. Our findings have practical implications for improving the effectiveness of movement therapy in a wide range of patient groups, maintaining upper extremity function in old adults, and for maximizing athletic performance.

## Introduction

Healthy humans are able to place light and heavy objects in small and large target locations with remarkable accuracy. Motor flexibility, the ability of the nervous system to generate different joint angle combinations while keeping end-effector movement unaffected, allows such reaching movements to be successful under a broad range of physical and dexterity demands. However, the current literature focuses almost exclusively on how dexterity demand affects movement accuracy during reaching with little or no attention to how physical demand affects reaching accuracy (for an overview see [1]). Moreover, the primary focus in the literature is on the trajectory of the end-effector and not on how the joint angles are coordinated to meet accuracy demands. The present study aims to extend the dexterity data and examines how dexterity and physical demand, or the interaction between these two factors, affect motor flexibility during a reaching task performed by healthy young adults.

It has been established that physical and dexterity demands affect kinematics of reaching. A number of studies reported that movement speed decreases as dexterity demand increases [1,2], demonstrating a speed-accuracy trade-off, a phenomenon most often quantified by Fitts’ law [1–3]. These studies show that humans are able to point at a target accurately because they slow the movement as they approach the target. This slowing allows the operator to rely on visual feedback and maintain the accuracy of the pointing movement [1,4–6].

In contrast, there are inconsistent findings concerning the effects of physical demand on kinematics during reaching. In one study the peak angular velocity decreased as physical demand (weight in the hand) increased during vertical arm movements [7]. However, in another study the time needed to achieve end-point peak velocity increased with increasing physical demand (larger resistance to movement) during a reciprocal aiming task [8]. The findings do not permit us to determine if a variation in load, a shift in motor control strategy, or some combinations of the two produced the kinematic changes and allowed participants to complete the task with similar accuracy independent of physical demand.

The present study is an attempt to resolve these inconsistencies. We explore the idea that humans retain movement accuracy during reaching independent of physical and dexterity demand by making small and coordinated adjustments in joint positions of the moving limb. The hypothesis is that co-variation between joints of the moving limb underlies flexibility in motor behaviour and mediates the preservation of movement accuracy independent of task difficulty. Such flexibility in movement execution is beneficial because it affords the effector system with a larger range of possible motor solutions to complete the reaching task [9]. A larger range of possible motor solutions improves the ability to rapidly adapt to the changing environmental and task constraints while keeping performance stable [10–14].

However, this idea is not in line with some previous findings because flexibility in joint coordination actually decreased in contrast to our suggestion of an increase with varying dexterity demands during reaching [6,15]. One reason for the discrepancy between our current hypothesis and past findings could be that the earlier reported variance analysis did not include all of the potentially relevant joints. Therefore, compensations at joints not included in the analysis could have affected motor flexibility [6]. Further, in a previous study subjects were instructed to reach for targets of different sizes at a constant speed, negating the speed-accuracy relationship [15]. Supporting our present hypothesis are the motor flexibility data recorded during walking, showing that coordination among leg joints increased when subjects were instructed to place their foot on the ground with high precision during gait [16].

Concerning the effects of physical demand on motor flexibility in lower extremity tasks using UCM analyses, it was suggested that an increase in motor flexibility compensated for the age-related weakness during the sit-to-stand task performed by old adults [17]. However, that study did not systematically manipulate physical demand. In addition, perhaps other factors such as impaired timing, balance, rate of torque generation [18–20] and not physical demand per se, contributed to the higher motor flexibility observed in old compared with young adults.

In total, there is conflicting and inconclusive evidence as to how dexterity and physical demand each and perhaps in an interactive manner would affect motor flexibility in general and during a reaching task in particular. Here, we examine the possibility that an increase in motor flexibility mediates the preservation of movement accuracy as physical and dexterity demands increase during a reaching task. We address this hypothesis by subjecting joint coordination patterns of the upper extremity to a UCM analysis [9]. The UCM analysis allows us to decompose variability in the effector system into variability stabilizing the end-effector position (goal-equivalent (GEV)) and variability de-stabilizing the end-effector position (non-goal-equivalent (NGEV)) during reaching. Larger GEV as compared to NGEV implies that the neuromuscular system employs flexible motor coordination patterns. We hypothesize that a) motor flexibility increases as physical demand increases; b) motor flexibility increases as the dexterity demand increases; c) motor flexibility increases more with increasing physical demands during high as compared with low dexterity demand conditions, and d) the increase in motor flexibility is characterized by an increase in GEV but unchanged NGEV as physical and dexterity demands increase.

## Materials and Methods

### Participants

20 healthy young adults (8 males and 12 females, 24.3 ±2 years) participated in the study. Those healthy participants and right-handed subjects were included who had no neurological or musculoskeletal disorders in the neck, shoulder, arm or hand, affecting pointing performance and had normal or corrected to normal vision.

### Ethics statement

The ethics committee of the Center for Human Movement Sciences, University Medical Center Groningen approved the study that was conducted according to the principles expressed in the Declaration of Helsinki. Before the start of the study, each participant read and signed a written informed consent.

### Experimental set-up

**Fig 1:**
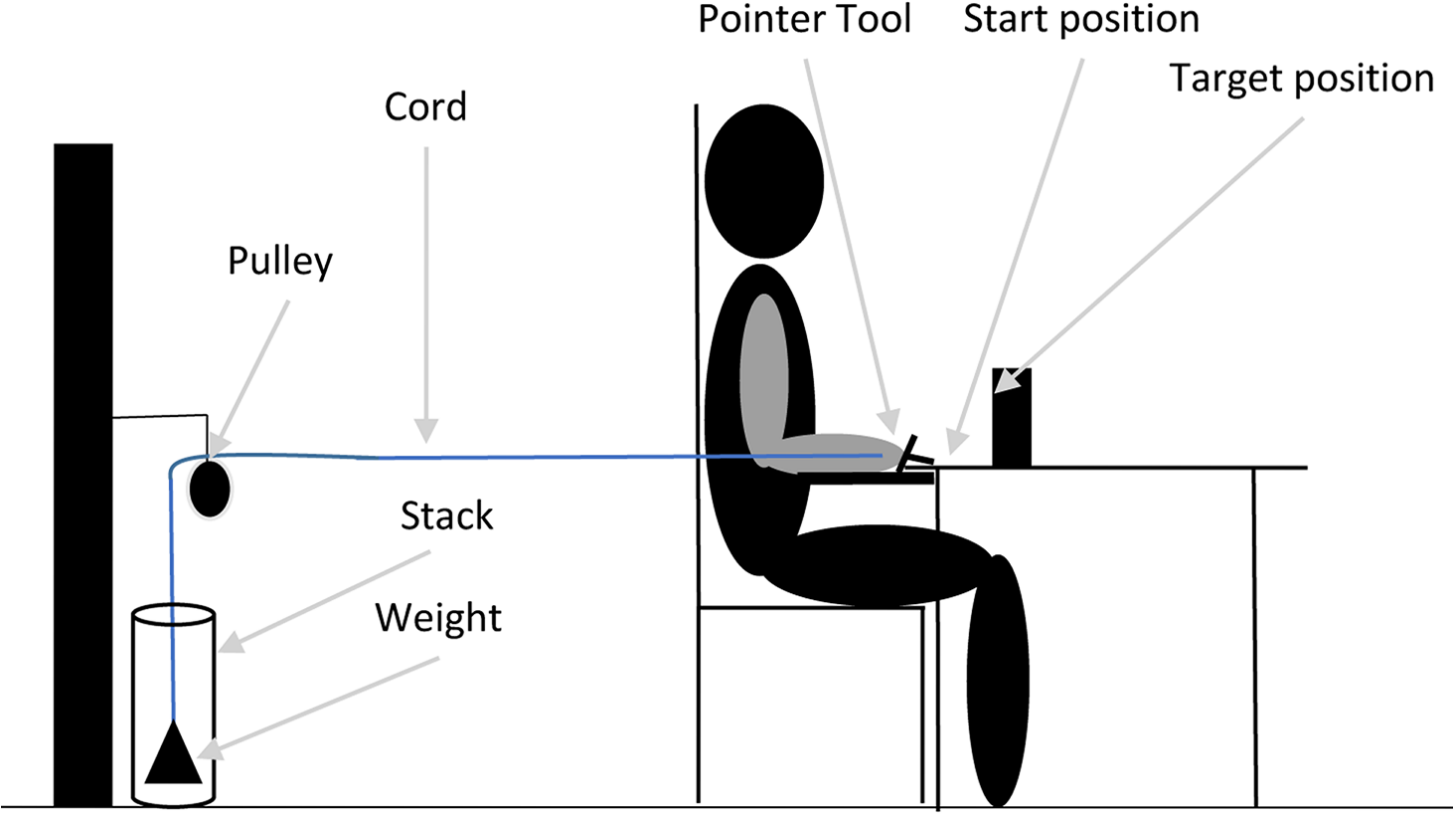
Experimental set-up.

The experimental set-up and manipulations were designed to determine the effects of dexterity and physical demands on flexibility in joint coordination patterns during upper extremity reaching. A 3D analysis of the right arm during a reaching movement was performed with an Optotrak motion capture system consisting of two units, sampling at 100Hz. Fig 1 shows a schematic overview of the experimental set-up. Subjects held a cross-like pointer and reached toward a target. The tool had a pointer tip of 2.5 cm length and 0.5 cm diameter that extended 5 cm from the second metacarpophalanegal joint when the pointer was held. A cord was attached to the posterior side of the pointer and was connected through a pulley to a weight stack (Fig 1). During the arm movement the cord was situated between the participants arm and body and did not interfere with the reaching movement. To collect the motion data, 6 triangular rigid bodies, each containing three LEDs, were placed on the participant’s sternum, right acromion, on the lateral aspect of the right upper arm just proximal to the insertion of the deltoid muscle, the lateral aspect of the right lower arm just proximal to the ulnar and styloid processus, the dorsal surface of the right hand and at the pointer tool [21].

Participants sat in an adjustable chair in front of a table so that the olecranon process with the elbow flexed at 90° was at the same height as the tabletop. The start posture was approximately 20° shoulder abduction, 90° elbow flexion and 90° pronation. To have a consistent start position, participants placed their right olecranon on an elbow support and the pointer tip in a pre-defined start position. The elbow support was positioned at the right side of the participants’ body at the same height with the table. The start position of the pointer tip was marked on the table with a dot of the size of the diameter of the pointer tip. During the start position the back of the pointer tool was placed against a wooden bar in order to release the load.

Five different weight conditions were used. In the no-weight condition a weight of 0.2 kg was attached to the cord, which was just enough to keep the cord taut.

### Experimental procedure

Before the start of the experiment, we measured each participant’s body mass, height, and handedness [22]. To quantify whether participants fatigued during the experiment, before and after the experiment subjects performed three trials of 4-s-long maximum voluntary contraction (MVC) against the load cell of a hand-held dynamometer (ErgoFet, Hoggan Health Industries, West Jordan, USA) in the start position of the pointing movement (as depicted in Fig 1). The average of the three trials was used in the analysis.

To minimize trunk movement during pointing, participants were stabilized with a crossover harness, tied to the chair. Before the start of each trial, participants’ position was checked and corrected as needed. The target position was at a distance of 25 cm (18.8 cm in depth and 16.6 cm vertical distance from table top) in front of the pointer tip.

Subjects heard a beep at the start and end of each pointing trial. Participants were instructed “to point as accurately and rapidly as possible to the target” after the beep and remain in contact with the target until a second beep occurred. After the second beep, participants moved their arm back to the start position. It was emphasized that the pointing task was not a reaction time task. If needed, participants were allowed to pause or rest at any time during the experiment.

### Experimental Design

Each participant performed 25 pointing trials to three different target sizes (1.56 cm,. 78 cm,. 39 cm) that resulted in targets with an index of difficulty (ID) of 4, 5 and 6 [3] and five different weight conditions (actual weights 0.2 kg, 0.7 kg, 1.2 kg, 1.7 kg and 2.2 kg, referred to as the 0 kg, 0.5 kg, 1 kg, 1.5 kg, 2 kg, load condition, respectively). The order of presentation of these blocks of 25 trials was randomized between subjects. Each participant performed 25 trials in 15 conditions resulting in 375 pointing trials. Subjects practiced the task 3 times before each new weight condition. There was one minute of rest between conditions.

### Joint angle computation

Joint angle computation followed the orientations from the ISB standardization proposal for the upper extremity by Wu et al (2005) [23]. Local coordinate systems were computed based on bony landmarks and the displacements of the markers on the rigid bodies. Based on the combination of the local coordinate systems, global and local orientations of segment coordinate systems were calculated [23]. For calibration, 17 bony landmarks were digitized with a standard pointer device [21]. The following joint rotations were computed: shoulder plane of elevation, shoulder elevation, shoulder inward–outward rotation, elbow flexion–extension, forearm pronation–supination, wrist flexion–extension and wrist abduction–adduction.

### UCM analysis

UCM analysis was performed as detailed previously [9,24–26]. The elemental variables were defined as the joint angular data of the shoulder, elbow and wrist resulting in a 7-DOF system. The pointer tip position (3 DOF) was selected as the performance variable. In order to relate changes in joint angles to changes in the position of the performance variable, the Jacobian (J) was computed based on a 3D forward kinematics model relating joint configurations to pointer tip position [27,28]. In an additional experiment we analyzed the accuracy of the forward kinematics model. The pointer tip was positioned for 3 seconds next to an Optotrak marker, which was fixated on a table. The root mean square of the difference between the 3D position of the modeled pointer tip position and the actual position of the Optotrak marker was 1.72 mm. Note that positioning the pointer tip on top of the Optotrak marker would have reduced the deviation but occluded the marker.

Based on the covariance matrix C of the joint configurations across trials, the variability components GEV and NGEV were computed by projecting the total variability (TOTV) in joint space to the null-space of J (null(J)^T^);GEV) and the orthogonal complement (orth(J^T^)^T^) (NGEV) by using Eqs 1–3:

TOTV=trace(C)/n 1.)

$$\text{NGEV}=\text{trace}(\text{orth}{\left(\text{J}\right)}^{\text{T}}*\text{C}*\text{orth}({\text{J}}^{\text{T}}))/\text{d}$$ 2.)

$$\text{GEV}=\text{trace}(\text{null}{\left(\text{J}\right)}^{\text{T}}*\text{C}*\text{null}(\text{J}))/\text{n-d}$$ 3.)

where n denotes the dimension of the joint space (n = 7) and d denotes the dimension of the task space (d = 3).

The column vectors of the matrices null(J)^T^ and orth(J^T^)^T^ form orthonormal bases for the null space of J and its’ orthogonal complement respectively. Each UCM component (TOTV, GEV and NGEV) was normalized by the number of DOF. To correct for non-normal data distribution all UCM components were log transformed before statistical analysis (GEV_T_ = log(GEV) and NGEV_T_ = (log(NGEV)). In order to quantify the strength of the stabilizing effect of motor flexibility, we computed the log transformed V_Ratio_ (V_RatioT_ = log(GEV/NGEV)) [26]. A V_RatioT_ > 0 implies that the end-effector position is a controlled variable [26].

### Individual joint variability and multi-joint covariation

One caveat of the UCM analysis is that it does not differentiate between UCM effects due to individual joint variability or multi-joint covariation. In order to determine whether UCM effects originated from multi-joint covariation and were not confounded by individual joint variability, a permutation analysis was performed [25,26]. Within the permutation analysis the UCM analysis is repeated as explained previously but with a covariance matrix where all covariation among joints is removed by setting the off-diagonal terms of the original covariance matrix (C) to zero (C_Perm_) [26]. After performing UCM analysis with C_Perm_ as covariance matrix we computed a surrogate data set of the V_Ratio_, V_RatioPerm_. V_RatioPerm_ contains the same amount of individual joint variability as in the original data set (V_Ratio_) but does not contain multi-joint co-variation. Larger amounts of V_Ratio_ as compared to V_RatioPerm_ imply that the UCM effects largely originated from multi-joint co-variation [25,26]. As for V_Ratio_, V_RatioPerm_ was log transformed before statistical analysis (V_RatioPermT_ = log(V_RatioPerm_)).

### Data analysis

The data were analyzed using customized MATLAB programs (Version R2012, Natick, USA). Coordinate data of each marker of the rigid bodies were filtered using a bi-directional 4th order Butterworth filter with a cut-off frequency of 8 Hz. Start and end of the movement was defined as the velocity of the pointer tip in forward direction above 2 mm·s^-1^ and below 2 mm·s^-1^. Based on the initiation and end of the movement the total movement time of each reaching trial was computed. For the UCM analysis during movement execution each reaching trial was time normalized and the UCM components GEV and NGEV, V_Ratio_ and CoV Index were partitioned into four phases (1–25%, 26–50%, 51–75% and 76–100%). For the UCM analysis at the end point of reaching, the last point of each reaching trial was used for the analysis. The tangential velocity of the pointer tip was computed based on the 3D position data and used to compute the symmetry index (time to peak velocity (s)\total movement time (s)) and peak velocities (m·s^-1^) of each reaching trial. The tangential end-effector position was computed as the sum of the square roots of the 3D position data.

### Statistical analysis

We used SPSS 20.0 and tested the hypotheses with four repeated measures ANOVA on variability per DOF during movement and at the end-point of the reaching movement. Hypothesis a), b) and c) were investigated with a repeated measures ANOVA on V_RatioT_ with phase (1–25%, 26–50%, 51–75% and 76–100%; during movement), dexterity demand (ID 4, ID 5, ID 6) and physical demand (0 kg, 0.5 kg, 1 kg, 1.5 kg, 2 kg) as within subjects factor. We tested hypothesis d) using a repeated measures ANOVA on variability per DOF with UCM component (GEV_T_ and NGEV_T_), phase (during movement), dexterity demand and physical demand as within subjects factor. This analysis allowed us to determine if physical and dexterity demands affected the variability components (i.e., larger GEV and no change in NGEV). We performed a repeated measures ANOVA on variability per DOF with Ratio component (V_RatioT_ and V_RatioPermT_), phases (during movement), dexterity demand and physical demand as within subjects factor to determine whether UCM effects originated from multi-joint covariation or from individual joint variability. To determine if behavior of the participants in the current study was similar reported by other studies that manipulated dexterity demand and physical demand, we quantified the effect of task demand on end-point kinematics. We conducted a repeated measures ANOVA on the standard deviation of the end-effector position, total movement time, duration acceleration time, duration deceleration time, symmetry index and peak velocity with dexterity demand and physical demand as within subjects factor. If the assumption of sphericity was violated, the Greenhouse-Geisser correction was applied. To interpret the significant effects of the ANOVA’s, the generalized eta-squared for effect size was used [29,30]. The effect sizes were interpreted according to Cohen’s (Cohen 1988) recommendation of. 02 for a small effect,. 13 for a medium effect and. 26 for a large effect [29].

To determine if motor flexibility was associated with accuracy of the end-effector position, we performed a correlation analysis. Associations during reaching were investigated for each movement phase between V_RatioT_ and the across trial standard deviation of the tangential end effector position. Furthermore V_RatioT_ during the last phase of the reaching movement and at the end-point of reaching was correlated with the effective target width (standard deviation·1.96). For this analysis the across trial standard deviation and V_RatioT_ of each participant was averaged within each movement phase across physical and dexterity demand conditions.

## Results

### Group-characteristics and strength profile

All recruited participants completed the experiment. Each participant performed 25 reaching trials for each condition of which on average 22.6 (±1.6) trials for each participant and condition were included in the data analysis. Trials were removed if one or more markers were invisible during the reaching movement. Table 1 shows the anthropometric and strength measurements. The experiment resulted in 3% decline in arm strength MVC, suggesting that fatigue did not affect the data.

**Table 1:** Anthropometric and strength data (N = 20).

		Mean	±SE	
Age (years)		24.3	2.0	
BMI (kg/m^2)		22.6	2.7	
Body Weight (kg)		72.2	13.2	
Height (cm)		178.9	10.0	
MVC (N/kg)	Pre	2.34	0.8	
	Post	2.27	0.8	

BMI, body mass index; MVC, maximal voluntary contraction measured in the start position of the pointing movement

### Joint position data

**Fig 2:**
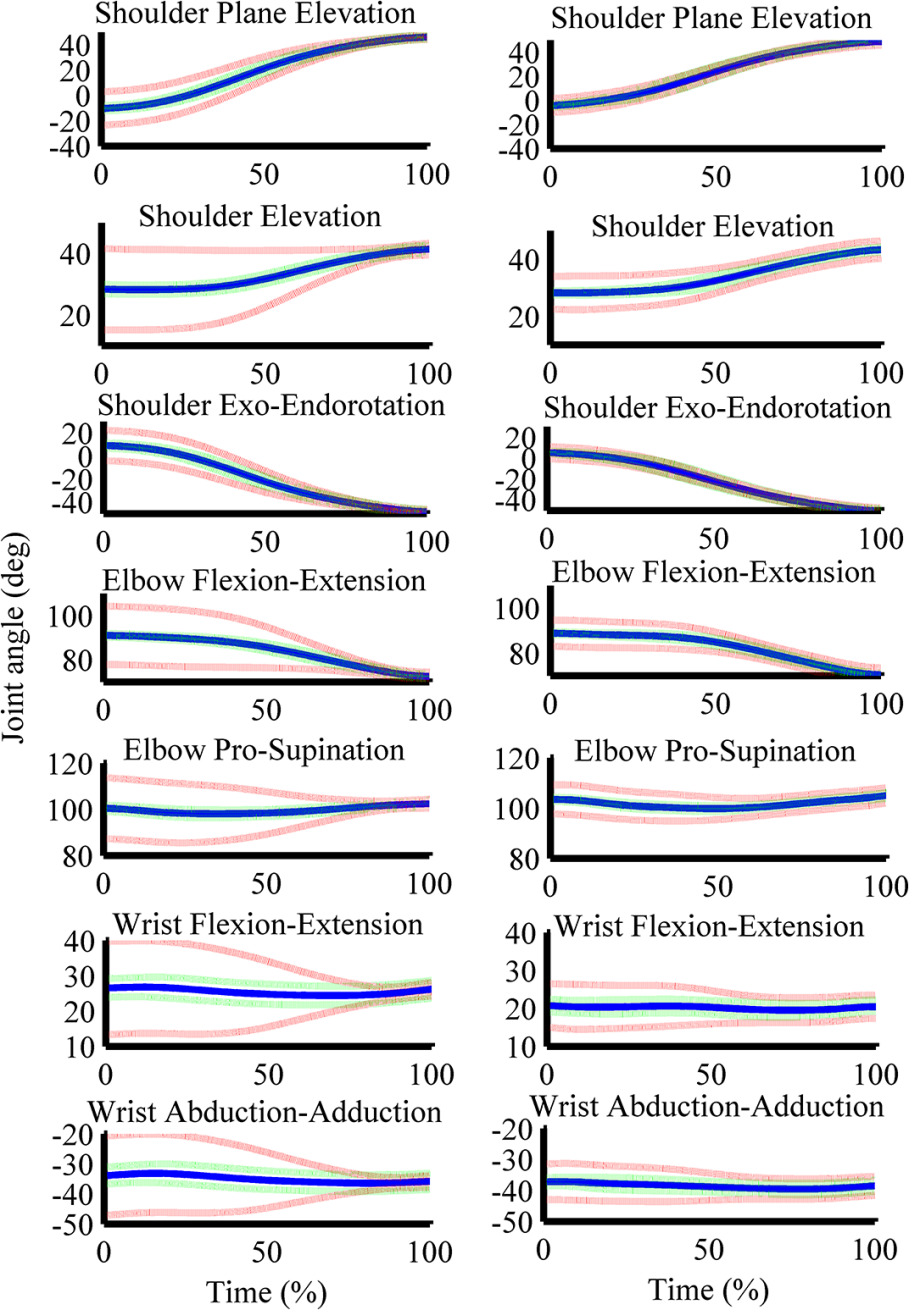
Time normalized joint position data. The blue line gives the mean, the dashed green line gives the mean of the within participant standard deviation and the red dashed line gives the standard error of the mean of the time normalized joint position data in degrees of the shoulder, elbow and wrist joint. The left panel gives the time normalized joint position data of the ID 4 and 0 kg condition and the right panel of the ID 4 and 2 kg condition.

Fig 2 shows the joint position data of the low dexterity demand and low physical demand condition (ID 4 and 0 kg; left panel) and the low dexterity demand and high physical demand condition (ID 4 and 2 kg; right panel). Joint excursions and within standard deviations appear similar across conditions. Fig 2 further shows that the within subject variance in joint position data was similar between conditions but the between subject variance in joint position data was lower in the ID 4 and 2 kg condition as compared to the ID 4 and 0 kg condition. The speed profiles of the end-effector were similar as compared to earlier studies [5,7,31–33].

### Flexibility in joint coordination patterns

**Fig 3:**
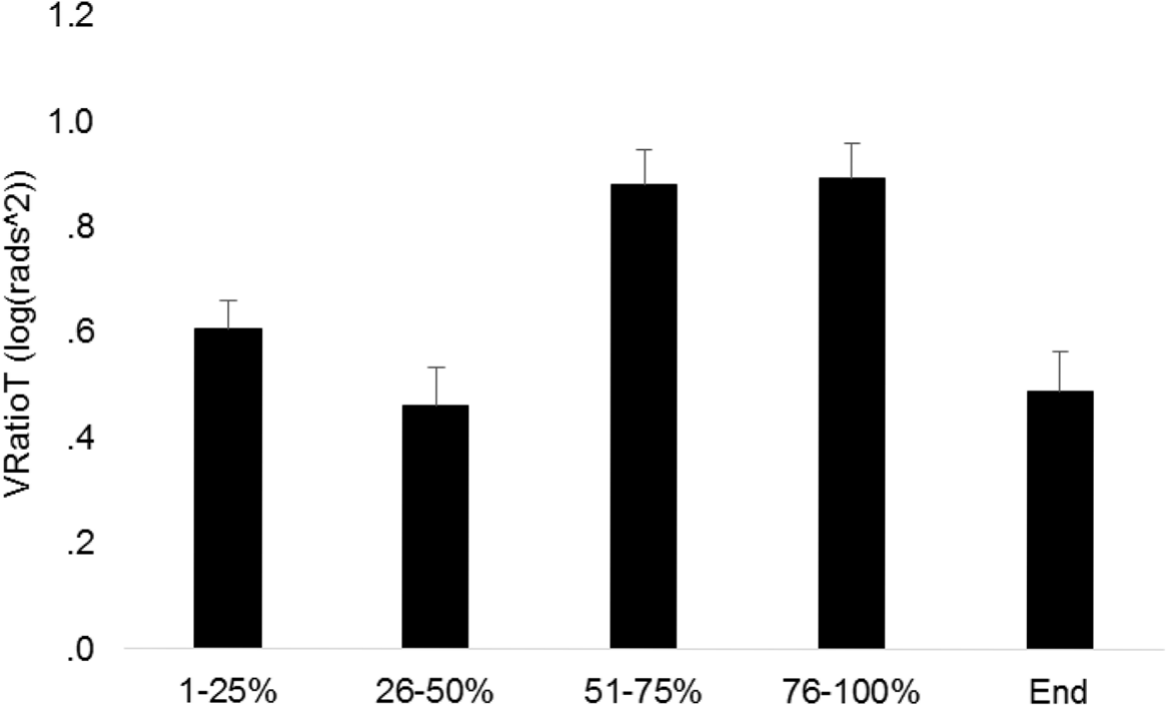
Log transformed V_Ratio_ (V_RatioT_) averaged across ID and physical demand conditions for all phases of the reaching movement and at the end-point of reaching. Vertical bars denote standard error of the mean.

Tables 2–5 present all significant and relevant non-significant results of the repeated measures ANOVA for each hypothesis during movement execution and at the end-point of reaching. The repeated measures ANOVA on V_RatioT_ during movement revealed a significant main effect for phases but not for physical or dexterity demand during movement execution and at the end-point of reaching (Tables 2 and 3). Fig 3 shows that the V_RatioT_ was highest during 51–100% of the reaching movement and lowest during 26–50% and at the end-point of reaching.

**Table 2:** Effects of movement phase, dexterity, and physical demand on V_RatioT_ during movement.

Within-subject factor		Mean	SEM	F	df	p-value	η^2^_G_
Phase	1–25%	. 60	. 054	18.1	1.7, 32	<.001	.12
	26–50%	. 46	. 072				
	51–75%	. 88	. 066				
	76–100%	. 89	. 066				
Dexterity	ID 4	. 72	. 052	. 3	2, 38	. 735	<.01
	ID 5	. 70	. 056				
	ID 6	. 71	. 050				
Physical	0 kg	. 72	. 053	. 9	4, 76	. 489	<.01
	0.5 kg	. 73	. 056				
	1.0 kg	. 68	. 065				
	1.5 kg	. 74	. 054				
	2.0 kg	. 67	. 058				

**Table 3:** Effects of dexterity and physical demand on V_RatioT_ at the end-point of reaching.

Within-subject factor		Mean	SEM	F	df	p-value	η^2^_G_
Dexterity	ID 4	. 56	. 085	1.3	2,38	. 297	<.01
	ID 5	. 47	. 101				
	ID 6	. 44	. 073				
Physical	0 kg	. 60	. 010	1.1	4,76	. 373	. 02
	0.5 kg	. 44	. 013				
	1.0 kg	. 56	. 015				
	1.5 kg	. 43	. 015				
	2.0 kg	. 43	. 015				

**Table 4:** Main and interaction effects of variability, movement phase, physical and dexterity demand on variability per DOF during movement.

Within-subject factor		Mean	SEM	F	df	p-value		η^2^_G_
Variability	GEV_T_	-6.53	. 09	213.8	1, 19	<.001		. 23
	NGEV_T_	-7.24	. 09					
Phase	1–25%	-7.18	. 10	40.2	1.9, 36.7	<.001		. 14
	26–50%	-6.51	. 09					
	51–75%	-6.72	. 08					
	76–100%	-7.10	. 10					
Physical	0 kg	-7.12	.074	22.8	2.6, 50.2	<.001		. 05
	0.5 kg	-7.00	.084					
	1.0 kg	-6.86	.098					
	1.5 kg	-6.77	.086					
	2.0 kg	-6.67	.102					
Variability x Phase				18.7	1.3, 32.1	<.001		. 02
Phase x Dexterity				2.97	3.2, 61.2	. 035		<.01

**Table 5:** Effects of variability and physical demand on variability per DOF at the end-point of reaching.

Within-subject factor		Mean	SEM	F	df	p-value		η^2^_G_
Variability	GEV_T_	-6.78	. 11	41.8	1, 19	<.001		.12
	NGEV_T_	-7.27	. 11					
Physical	0 kg	-7.27	. 10	11.3	4, 76	<.001		.04
	0.5 kg	-7.08	. 11					
	1.0 kg	-6.99	. 13					
	1.5 kg	-6.94	. 12					
	2.0 kg	-6.83	. 11					

Analysis on the UCM components during movement execution revealed significant main effects for variability, phases and physical demand and significant interaction effects between variability and phase and between dexterity demand and phase (Table 4). At the end point of the reaching task analysis revealed a significant main effect for variability and physical demand (Table 5).

**Fig 4:**
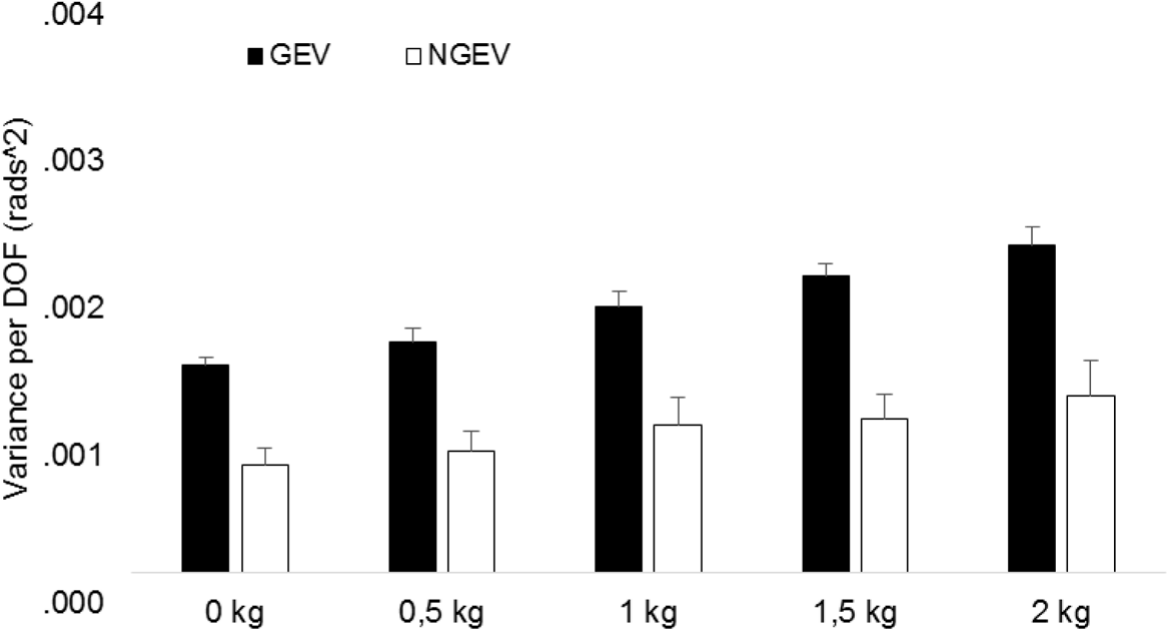
GEV and NGEV averaged across ID and movement phases for the physical demand conditions. Vertical bars denote standard error of the mean. Note that the statistical analysis was performed on the log transformed GEV (GEV_**T**_) and NGEV (NGEV_**T**_).

**Fig 5:**
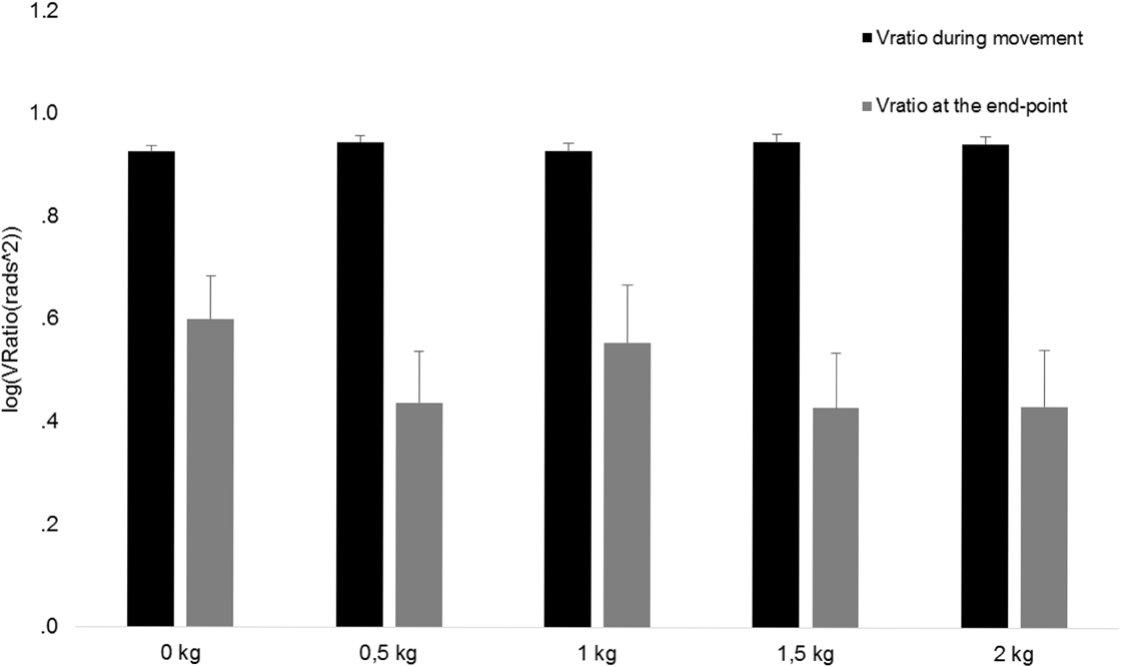
Log transformed V_Ratio_ (V_RatioT_) averaged across ID and movement phases for the physical demand conditions. Vertical bars denote standard error of the mean.

The main effect for variability showed that the amount of GEV was significantly larger than NGEV during all phases and at the end-point of the reaching movement (Tables 4 and 5). This finding implies that the end-effector position was a controlled variable during reaching [9]. The significant main effect for phase during movement demonstrated that the average amount of variability ((GEV_T_ + NGEV_T_)/2) in elemental variables was largest during 26–75% of the reaching movement (Table 4). As illustrated in Fig 4 the main effect of physical demand shows that GEV and NGEV proportionally increase with increasing physical demands (Fig 4). This finding and the results from the analysis on V_RatioT_ demonstrate that the stabilizing effect of motor flexibility was maintained despite increases in NGEV and GEV with increasing physical demands (Fig 5; Tables 2 and 3). In other words, although the variability measures GEV and NGEV increased with increasing resistance to the reaching movement their relative values did not change.

The significant interaction between variability and phase was further explored with post-hoc analysis on GEV_T_ and NGEV_T_ averaged across ID and physical demand conditions. GEV_T_ and NGEV_T_ values of each phase were compared with the preceding phase, demonstrating that after Bonferroni correction all these phases differed for NGEV_T_ but for GEV_T_ the phases differed only between 1–25% and 26–50% and between 51–75% and 76–100% (all p-value’s <. 001).

### Individual joint variability and multi-joint co-variation

**Fig 6:**
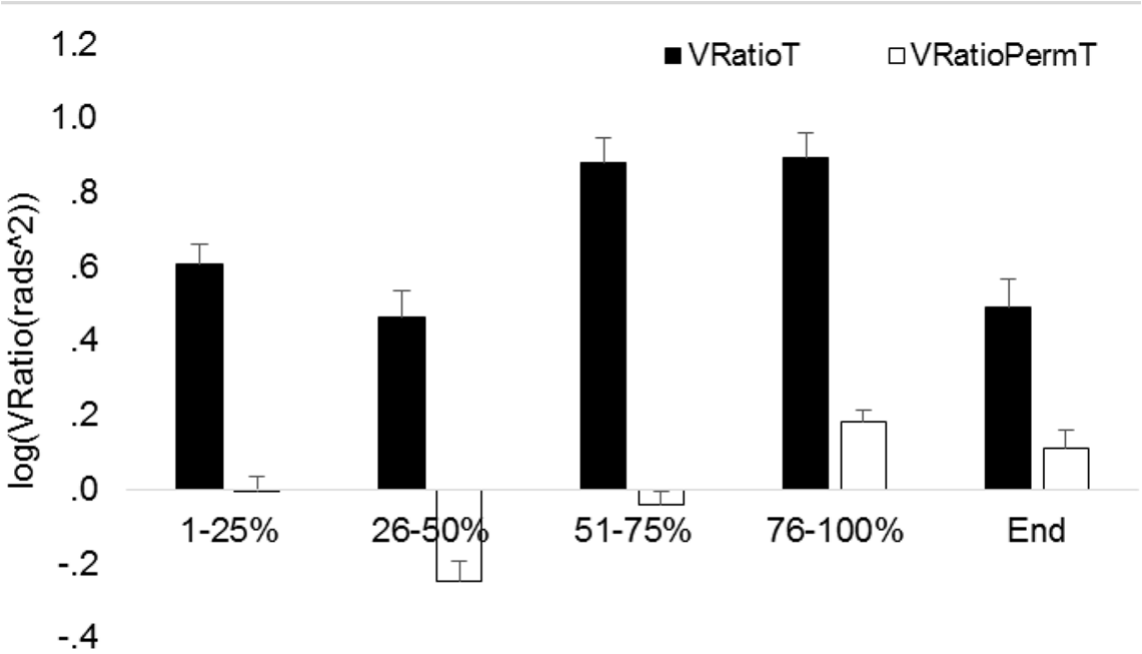
Log transformed V_Ratio_ (V_RatioT_) and V_RatioPerm_ (V_RatioPermT_) averaged across ID and physical demand conditions for all phases of the reaching movement and at the end-point of reaching. Vertical bars denote standard error of the mean.

**Fig 7:**
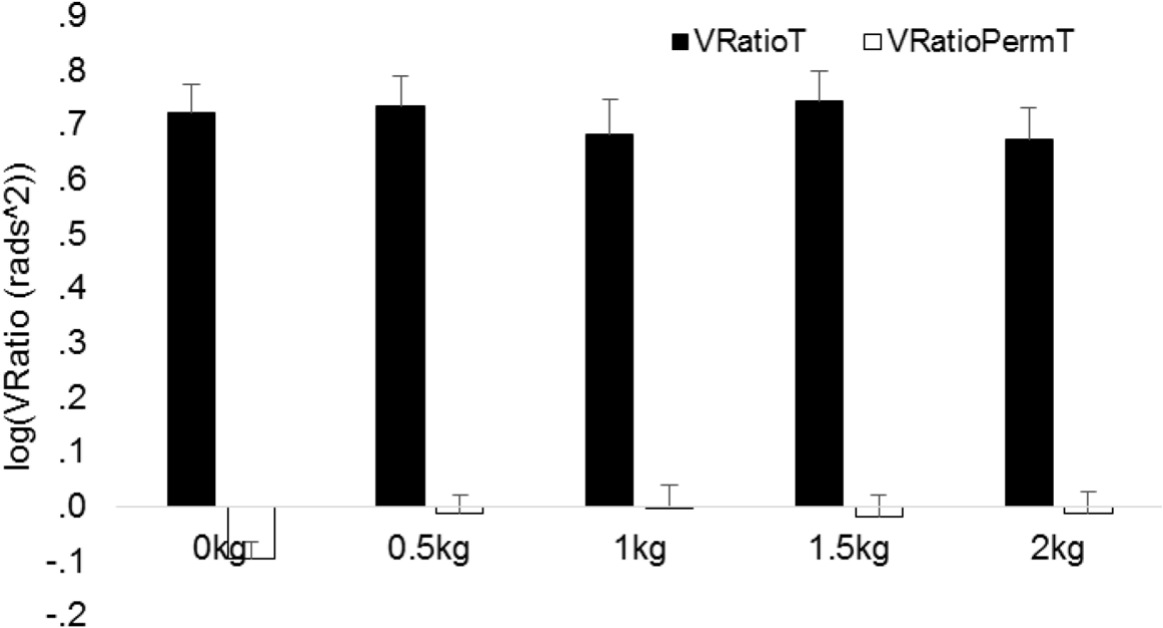
Log transformed V_Ratio_ (V_RatioT_) and V_RatioPerm_ (V_RatioPermT_) averaged across ID and movement phases for all physical demand conditions. Vertical bars denote standard error of the mean.

In order to determine whether UCM effects originated from multi-joint covariation or individual joint variability we performed a repeated measures ANOVA on ratio components (V_RatioT_ and V_RatioPermT_) with phase, dexterity demand and physical demand as within subjects factor. The analysis during movement revealed a significant main effect for Ratio component and phases and significant two-way interaction effects between ratio component and phases and between ratio component and physical demand (Table 6). Figs 6 and 7 show that V_RatioT_ was larger than V_RatioPermT_ during all phases and physical demands indicating that UCM effects largely originated from multi-joint covariation and not from individual joint variability.

**Table 6:** Main and interaction effects of ratio, movement phase and physical demand on ratio components (V_Ratio_ and V_RatioPerm_) during movement.

Within-subject factor		Mean	SEM	F	df	p-value		η^2^_G_
Ratio	V_RatioT_	. 71	. 05	336.6	1, 19	<.001		. 45
	V_RatioPermT_	-.03	. 03					
Phase	1–25%	. 30	. 10	22.8	1.6, 31.2	<.001		. 08
	26–50%	. 11	. 09					
	51–75%	. 42	. 08					
	76–100%	. 54	. 10					
Ratio x Phase				15.8	1.9, 35.6	<.001		. 01
Ratio x Physical				3.1	2.9, 55.8	. 034		<.01

The significant interaction effects were further investigated with post-hoc analysis. The significant interaction between Ratio component and phase was further explored with post-hoc analysis on V_RatioT_ and V_RatioPermT_ averaged across ID and physical demand conditions. V_RatioT_ and V_RatioPermT_ of each phase were compared with the preceding phase, demonstrating that after Bonferroni correction all these phases significantly differed for V_RatioPermT_ (all p-values’ <. 001) but for V_RatioT_ the phases differed only between the phases 1–25% and 26–50% (t_19_ = 3.401; p =. 003) and between the phases 26–50% and 51–75% (t_19_ = -6.2; p <. 001). As illustrated in Fig 6 this finding implies that the amount of individual joint variability relative to the amount of V_Ratio_ was largest during the last phase of the reaching movement. The significant interaction between Ratio component and physical demand was further explored with post-hoc analysis on V_RatioT_ and V_RatioPermT_ averaged across ID and phases. Each physical demand condition was compared with the 0 kg condition demonstrating that after Bonferroni correction V_RatioT_ did not differ across physical demands but V_RatioPermT_ of the 0 kg condition was significantly lower as compared to the 0.5 kg condition (t_19_ = - 2.8; p =. 011). This finding implies that the amount of individual joint variability relative to the amount of V_Ratio_ was larger in the 0.5 kg as compared to 0 kg condition.

### End-effector kinematics

In order to determine whether behavior of the participants in the current study complied to findings in other studies we determined the effect of task demand on end-point kinematics. Table 7 shows the significant main effects of the repeated measures ANOVA on movement time and peak velocity of the end-effector for the dexterity demand and physical demand conditions. As expected the total movement time increased and peak velocity values decreased as dexterity and physical demands became more challenging (Table 7). The mean coefficient of the regression line between movement time (s) and ID, representing the speed-accuracy trade-off for the dexterity demand conditions was. 035 (±.025). Furthermore, kinematic analysis of the end-effector showed that the symmetry index significantly increased as physical demands increased (F_4, 76_ = 62.9; p = <.001; η^2^_G_ =. 19). Further analysis on the duration of the acceleration and deceleration showed that both the acceleration and deceleration prolonged with higher dexterity (acceleration: F_1.4,27.4_ = 7.6; p =. 005; η^2^_G_ =. 02; deceleration: F_1.3, 24.3_ = 5.3; p =. 023; η^2^_G_ =. 03) and physical demands (acceleration: F_1.7, 33.1_ = 100.9; p <. 001; η^2^_G_ =. 32; deceleration: F_2.9, 55.5_ = 3.7; p =. 018; η^2^_G_ <. 01). The effect sizes show that the acceleration phase was more affected by the physical as compared to the dexterity demand and the deceleration phase was more affected by the dexterity as compared to the physical demand.

**Table 7:** Effects of dexterity and physical demand on end-effector kinematics.

Within-subject factor				Mean	SEM	F	df	p-value	η^2^_G_
Movement time (sec.)	Dexterity		ID 4	.541	.034	6.8	1.3, 25.1	. 010	.03
			ID 5	.576	.036				
			ID 6	.611	.036				
	Physical		0.0 kg	.515	.029	51.5	2.1, 40.5	<.001	.07
			0.5 kg	.547	.035				
			1.0 kg	.570	.033				
			1.5 kg	.611	.036				
			2.0 kg	.636	.038				
Peak Velocity (m/sec.)	Dexterity		ID 4	1.014	.051	6.1	1.2, 23.3	.005	.13
			ID 5	.959	.046				
			ID 6	.924	.042				
	Physical		0.0 kg	1.046	.046	44.4	2, 37.7	<.001	.26
			0.5 kg	1.000	.048				
			1.0 kg	.959	.045				
			1.5 kg	.926	.043				
			2.0 kg	.897	.041				

### End-effector accuracy

**Fig 8:**
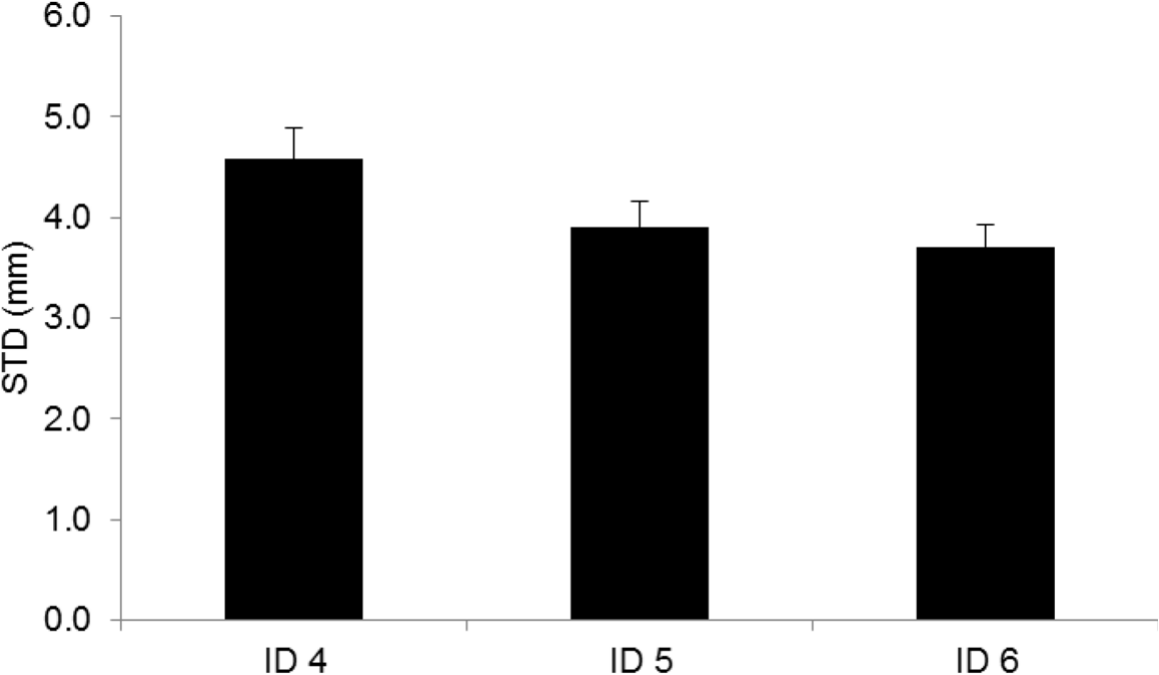
Effects of dexterity demand on end-effector accuracy expressed as standard deviation error in mm. * p =. 05. Vertical bars denote standard error of the mean.

The repeated measures ANOVA on standard deviations of the tangential end-effector position revealed a significant main effect for dexterity demand (F_2,38_ = 13.4, p <. 001, η^2^_G_ =. 172) and physical demand (F_4,76_ = 5.9, p <. 001, η^2^_G_ =. 070). As expected, Fig 8 shows that as the dexterity demand increased standard deviations of the end-effector position decreased. Pairwise comparisons showed that after Bonferroni correction the standard deviation of the tangential end-effector position was significantly lower for the ID 5 (p =. 002) and ID 6 (p =. 004) condition compared to the ID 4 condition.

**Fig 9:**
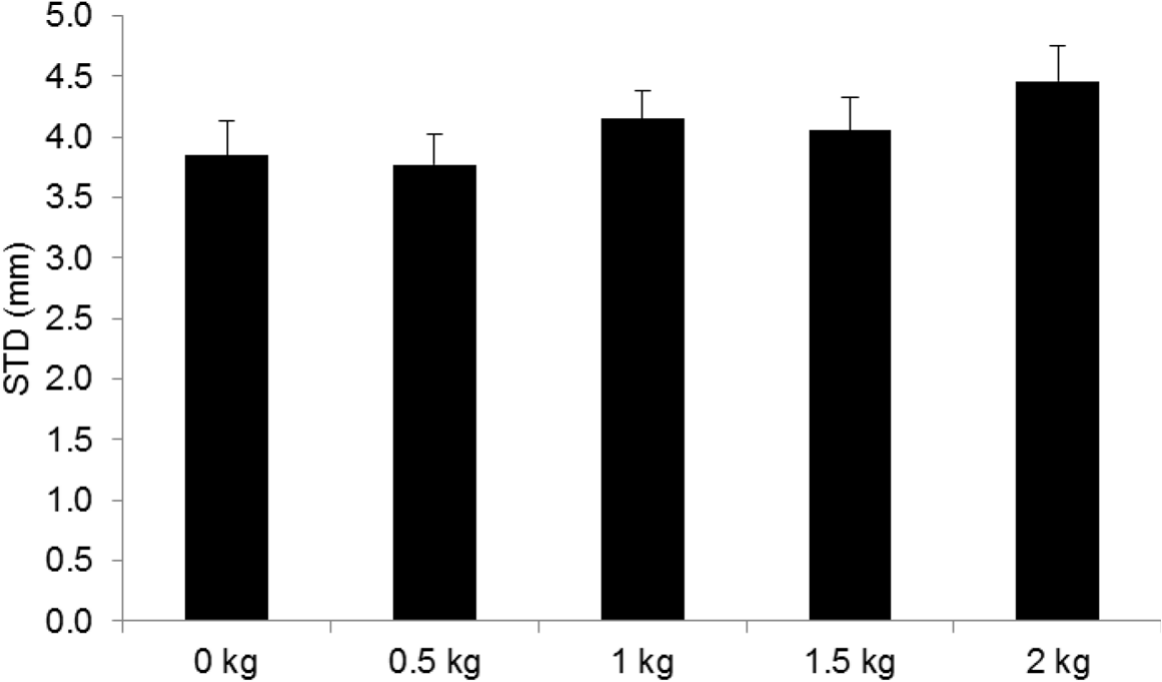
Effects of physical demand on end-effector accuracy expressed as standard deviation error in mm. * p =. 05. Vertical bars denote standard error of the mean.

Fig 9 illustrates the significant main effect of the physical demand on end-effector accuracy. Pairwise comparisons showed that after Bonferroni correction the standard deviation of the tangential end-effector position was only significantly higher during the 2 kg condition compared to the 0 kg physical demand condition (p =. 019). Comparing the 0 kg condition with the 0.5, 1 kg and 1.5 kg condition did not show any significant difference.

Additionally we investigated whether participants did reach the target by calculating the effective target width of the tangential end-effector position. The effective target width for the ID 4 (1.56 cm) condition was. 89 (±.58) cm, for the ID 5 (.78 cm) condition. 76 (±.52) cm and for the ID 6 (.39 cm) condition. 72 (±. 46) cm. This demonstrated that for ID 5 and ID 6 the targets were often missed.

### Associations between motor flexibility and end-effector accuracy

Correlation analysis revealed significant negative associations between V_RatioT_ and the across trial end-effector standard deviation of the last movement phase (75–100%; r = -.461; DF = 18; p =. 041) and between V_RatioT_ of the last movement phase and the effective target width (r = -.634; DF = 18; p =. 003). Interestingly, V_RatioT_ at the end point of reaching was not associated with the effective target width (r = -.110; DF = 18; p =. 644).

## Discussion

The current study examined the idea that an increase in motor flexibility mediates the preservation of movement accuracy as physical and dexterity demands increase during a reaching task. We hypothesized a) motor flexibility increases as physical demands increase; b) motor flexibility increases as dexterity demands increase; c) motor flexibility increases more with increasing physical demands during high as compared to low dexterity demand conditions, and d) the increase in motor flexibility would be characterized by an increase in GEV while NGEV remains rather unaffected with higher physical and dexterity demands. Our results showed that despite increases in GEV and NGEV as a function of physical demand (Fig 4), V_Ratio_ remained unchanged as the physical demand of the reaching task increased (Fig 5). Increase in the dexterity demand did not affect the amount of GEV or NGEV. Our findings show that the neuromuscular systems’ behavior is affected by changes in the physical but not dexterity demand during upper extremity reaching. We argue that as physical demand increases, larger sensorimotor noise in the system causes an increase in NGEV. The neuromuscular system adapts to these changes and increases the exploration of joint coordination patterns stabilizing the end-effector position (i.e., GEV) to maintain stable values of V_Ratio_. This strategy allows the preservation of movement accuracy despite larger physical demands. Because there was no interaction between physical and dexterity demand, we discuss these two effects on motor flexibility separately. Our findings are in line with a previous study that examined the pattern of adaptation to a force-field applied orthogonal to movement direction of a planar reaching task [34]. Despite the question and experimental design of that paper was different from that of ours, there are some similarities in the findings that both GEV and NGEV were higher as the movement was perturbed by the force-field [34].

### Motor flexibility is maintained as physical demands increase during reaching

The results demonstrate a proportional increase of GEV and NGEV without a change in V_Ratio_ as physical demands increase during reaching (Figs 4 and 5). This finding suggests that the neuromuscular system increases the exploration of those joint angle combinations stabilizing the pointer tip position (GEV) proportional to the increase in de-stabilizing variability (NGEV) during physically more demanding reaching conditions. This strategy allows the neuromuscular system to maintain stable values of V_Ratio_ and preserve movement accuracy despite larger physical demands. Overall our findings do not confirm our hypothesis but agree with the idea that under certain conditions when the physical demand is high during a motor task flexibility in the available DOF is employed to counter the high physical demands and mediate performance stability [17]. Furthermore dexterity and physical demands did not interact during reaching. A lack of effect of dexterity on motor flexibility is especially surprising because it is reasonable to expect motor flexibility to adapt to target size.

To the best of our knowledge this is the first demonstration of a relationship between physical demand and motor flexibility in a reaching task. We interpret this relationship in the context of previous studies showing that sensorimotor noise in the neuromuscular system increases approximately linearly with increasing force demands during motor tasks [35–37]. Sensorimotor noise interferes with the neural signal that produces the reaching movement. We argue that in our study the documented increase in NGEV with increasing force demands originated from demand-dependent increase in sensorimotor noise that is presumably present at each phase of a reaching task, including object localization, motor command generation, and muscle activation by this command to execute the movement [38]. The neuromuscular system counters the disturbing effects of increasing noise by a proportional increase in the exploration of those joint angle combinations stabilizing the end-effector position. This increase in GEV prevents a drop in the V_Ratio_ and allows the preservation of performance stability despite higher physical demands.

We documented that the neuromuscular system makes flexible use of the available DOF to mediate performance stability at relatively small increases in the physical demand during reaching. Old adults increased GEV and constrained NGEV to mediate performance stability during a physically high demanding sit-to-stand task [17]. That the neuromuscular system in young and old adults makes flexible use of the available DOF to mediate performance stability across a broad range of physical demands and across different tasks suggests that this may be a general strategy to maintain motor performance in the face of increasing physical demand. In line with this argument we found significant correlations between the strength of the stabilizing effect of motor flexibility (V_Ratio_) and accuracy of motor performance showing that those participants who employed larger V_Ratio_ were more accurate. Interestingly, this association was strongest between V_Ratio_ of the last movement phase and accuracy at the end-point of reaching and there was no association between V_Ratio_ at the end-point of reaching and end-point accuracy. The finding that this correlation between the stabilizing effect and end-effector accuracy was present in the last phase of the movement but not at the moment that the target was actually reached, made us treat our finding with caution. We think further study is required to characterize this relationship more accurately.

The effect of physical demand on motor behavior was previously investigated by analyzing how muscle activation patterns change during motor tasks with different loads [39–43]. These studies showed that agonist-antagonist muscle coactivation increased with increasing load instability or with changing external loads. The authors argued that as the load of the most distal segment changes (as in some conditions of our experiment), magnitude of interaction torques may increase and increases in agonist-antagonist muscle coactivation mediate performance stability by minimizing the disturbing effects of limb dynamics [42,44]. Intuitively, this concept of larger antagonist muscle coactivation with higher physical demands contrasts with our findings on the flexible use of the available DOF to mediate performance stability as physical demands increase. Linking the equilibrium point hypothesis (EPH) [45,46] with the UCM approach may resolve this discrepancy. Latash (2010) proposed that increasing antagonist muscle coactivation and flexibility in joint coordination patterns are two distinct motor control strategies mediating performance stability as the physical constraints of a motor task change [47]. Within this paradigm, it is possible that flexibility in the available DOF increases when muscle coativation increases. However detailed analyses of how motor flexibility and muscle coactivation are regulated concurrently is beyond the scope of the present paper but will be the topic of one of our future studies.

Although our general results are in line with our previous study using a lower extremity task [17], we did not document an increase but maintenance of the stabilizing effect of motor flexibility with increasing physical demands. GEV was significantly higher in old as compared to young adults and NGEV did not differ between age-groups [17]. We suspect that the diminished muscle reserve capacity in the whole-body sit to stand task as compared to our less demanding reaching task may underlie the GEV and NGEV differences. We argue that during less demanding reaching tasks the increase in NGEV with increasing physical demands is counteracted by a proportional increase in GEV. This strategy is sufficient to keep motor flexibility versatile and mediate performance stability during relatively small increases in the physical demand. However during high demanding tasks it is feasible to assume that the amount of GEV, which can be employed by the neuromuscular system to counter the increase in NGEV is not infinite. Therefore when operating close to maximum physical capacity, the employment of GEV is increased and NGEV is constrained. Furthermore, in contrast to the reaching task, instability of the performance manifested through larger NGEV in the complex sit-to-stand task could cause a fall and harm the integrity of the neuromuscular system. Constraining the increase of NGEV with increasing physical demands during postural tasks might therefore be a safety mechanism. Collectively the present and previous findings provide evidence for a task-dependent control of motor flexibility that is scaled to the reserve capacity of the system. Accordingly, neuromuscular system’s motor performance is stabilized through: a) a proportional increase in GEV as compared to NGEV during reaching tasks relatively far from the maximum load that can be handled and b) increases in GEV and constraining NGEV during high demanding postural tasks.

### End-effector kinematics and physical demand

As the resistance to the movement increased during physically more demanding conditions, peak velocity decreased and total movement time increased. Total movement time increased because the acceleration phase became longer, confirming previous findings. For example, movement times became longer when the inertial load increased during reaching tasks around the elbow joint in the horizontal plane [48]. Peak angular velocity also decreased as physical demand increased during vertical arm movements [7]. Finally there was an increase in the time needed to achieve peak velocity with increasing physical demand during a reciprocal aiming task in the horizontal plane [8].

### Motor Flexibility is unrelated to dexterity demand during reaching

In contrast to our hypothesis, there was no relationship between dexterity and motor flexibility during upper extremity reaching, suggesting that dexterity demand did not affect the exploration of joint angle combinations. Our results further showed that increase in dexterity demand affected reaching kinematics, that is, resulting in longer movement time and lower peak velocity. The documented longer movement time was characterized by a prolongation of the deceleration and acceleration phase. A prolongation of the deceleration phase is assumed to allow the operator to rely more on visual feedback [1,4–6]. These kinematic adaptations were in line with previous studies [1].

The finding that dexterity demand did not affect motor flexibility in combination with our results on end-effector kinematics and the well-documented speed accuracy trade-off in earlier studies [1], suggests that adjustments in end-effector kinematics rather than motor flexibility mediate movement accuracy as dexterity demands increase. However it is important to note that compared to a previous study [1], our results showed a less pronounced speed-accuracy trade-off and a smaller decrease in the effective target widths with higher dexterity demands (Table 7; Fig 8). During the highest dexterity demand condition (ID 6) many participants even missed the target. These findings imply that our participants chose a strategy of reaching fast at the expense of accuracy. This relative importance of movement speed over accuracy might explain in part that motor flexibility did not change with increasing dexterity demand during reaching.

Previous studies reported that the neuromuscular system employs less motor flexibility when reaching to targets with a high dexterity demand [15]. The specific experimental conditions might cause the discrepancy between our results and the previous data because in that study subjects were instructed to move at the same speed across trials and reached the target in the same time independent of ID [15]. In line with the Fitts’ task paradigm, we instructed our participants to move as rapidly and accurately as possible. Further, Tseng et al (2003) did not report end-point accuracy during either the high or low ID condition, which makes it unclear if participants actually reached the target during both dexterity demand conditions [15].

### Practical implications

When we extend our arm and place first a light then a heavy object at the same target location, the object’s higher mass increases the amount of sensorimotor noise causing an increase in de-stabilizing variability (NGEV) in the neuromuscular system, which makes accurate movement execution more challenging. To place the heavier object as rapidly and accurately as the light one, the neuromuscular system makes minute adjustments and adapts. There is opportunity for such adjustments because the object’s greater mass slows the movement. The UCM analysis in the present study captured such adjustments and revealed that even healthy young adults execute reaching with relatively high loads by unconsciously increasing the exploration of the range of available and possible motor solutions to complete the task according instructions: rapidly and accurately. Such findings have implications for exercise interventions designed to improve athletic performance and make upper extremity movements better timed and coordinated in a variety of patient groups and old adults.

Highly trained athletes such as basketball players are able to execute the same motor skills, like a jump shot, from many positions on the field and in unpredictable game situations. Such athletes possess an expansive movement repertoire that affords them performance flexibility. Our findings suggest that practicing a specific motor task like a jump shot, under physically challenging conditions by using a slightly heavier basketball would require ball players to unconsciously explore a larger range of possible motor solutions. Increasing the exploration of motor solutions which do not deviate the ball from its desired path (GEV) would allow the athlete to counter the larger de-stabilizing variability and perform an accurate jump shot even though the ball is heavier. A chronic exploitation to a larger range of possible motor solutions provides the athlete with more flexibility and the ability to choose from a larger range of motor solutions when the weight of the ball is reduced during a game.

In daily life, we perform the same tasks in different postural contexts and positions and, for example, reach for objects when we stand or sit. In contrast to healthy individuals, patients and old adults who suffer from upper extremity coordination deficits find even simple reaching tasks challenging and, similarly to athletes, would benefit from therapy that manipulates physical demand.

### Limitations

In the current study we investigated a reaching task with the upper extremity. However, due to the task manipulation participants held a pointer tool in their hand and the metacarpophalangeal and interphalangeal joints were excluded from the analysis. This resulted in 7 instead of 9 DOF for the geometric arm model, which limits comparability of our results to reaching or pointing tasks involving 9 DOF. Furthermore our experimental set-up did not account for individual differences in height and arm length. Therefore at the end of the reaching movement participants with shorter arm length had a smaller residual range of motion in the involved joints, which might have limited their ability to explore varying joint coordination patterns.

## Conclusion

We observed a relationship between physical demand and flexibility in the available DOF when young adults were instructed to point at a target rapidly and accurately. We interpret this finding in the context of previous studies showing that sensorimotor noise increases with increasing physical demands. The increase in sensorimotor noise with higher physical demands was represented in our study by larger NGEV. We propose that making flexible use of the available DOF to counter the de-stabilizing increase in NGEV is a general strategy employed by the neuromuscular system to preserve movement accuracy under physically more demanding conditions. The dexterity demand of the reaching task did not affect flexibility in the joint coordination patterns. Future studies should examine the possibility that next to adaptations in flexibility of joint coordination patterns, modulation of agonist-antagonist muscle coactivation is an additional and perhaps synergistic strategy contributing to accurate task execution under physically more challenging conditions.

## Supporting Information

S1 File Anthropometrics and UCM measures. The y-axis gives the subject numbers and the x-axis the age, height, weight, MVC measures and log transformed UCM measures (GEV, NGEV, Vratio) for each movement phase (1–25%, 26–50%, 51–75%, 76–100% and end-point) and dexterity (ID4, ID5 and ID 6) and physical demand (0 kg, 0.5 kg, 1 kg, 1.5 kg, 2 kg) condition.
(XLS)
